# Dietary antioxidants and micronutrient status in varicocele-associated male infertility: oxidative stress-mediated effects on semen quality

**DOI:** 10.3389/fnut.2026.1832900

**Published:** 2026-07-13

**Authors:** Bo Fang, Hongbin Lou, An Fu, Xiaoming Yi, Wei Zhang, Haowei He, Xuejun Shang, Wen Cheng, Dian Fu

**Affiliations:** 1 Department of Urology, Nanjing Jinling Hospital, Affiliated Hospital of Medical School, Nanjing University, Nanjing, China; 2 Department of Urology, Anhui Wannan Rehabilitation Hospital, Anhui, China

**Keywords:** antioxidants, fertility outcomes, male infertility, oxidative stress, sperm quality, varicocele

## Abstract

**Background:**

Varicocele causes male infertility through its association with oxidative stress that damages sperm. Dietary antioxidants together with micronutrients help to lower oxidative stress while enhancing sperm production, but their impact on varicocele-related infertility remains to be proven.

**Methods:**

The researchers conducted a retrospective cohort study of 900 men with varying degrees of varicocele, including 300 with Grade I, 400 with Grade II, and 200 with Grade III. The study collected data on participants’ demographics, body measurements, daily habits, food consumption, micronutrient levels, oxidative stress indicators, antioxidant enzyme activity, reproductive hormone levels, semen characteristics, and reproductive success. The study used multiple linear and logistic regression analyses to identify factors affecting sperm concentration and motility, DNA fragmentation, and spontaneous conception. The researchers used standardized beta coefficients to quantify the contribution of each main predictor variable to the results.

**Results:**

The negative effects on sperm concentration and motility are attributable to varicocele severity, age, BMI, smoking, reactive oxygen species (ROS), and malondialdehyde (MDA), which generate DNA fragmentation, thereby establishing statistical significance at *p* < 0.05. The dietary antioxidants vitamin C, vitamin E, zinc, and selenium had positive effects on sperm concentration (*β* = 0.09–0.18) and motility (*β* = 0.10–0.16), while they had negative effects on DNA fragmentation (*β* = −0.07 to −0.15). The regression models explained 40–42% of the variability in sperm parameters. The logistic regression analysis found that antioxidant consumption increased the probability of natural conception (OR 1.05–1.13, *p* < 0.01), whereas age, BMI, varicocele degree, smoking, and ROS and MDA reduced the probability of conception (OR 0.64–0.97, *p* < 0.05). Clinical outcomes tracked 30% of participants who achieved natural conception, while 25% underwent assisted reproductive technology (ART) cycles, and the group attained a live birth rate of 28%. The group with Grade I varicocele and higher antioxidant intake showed improved outcomes.

**Conclusion:**

Men with varicocele demonstrated impaired sperm quality and reduced fertility potential, which appeared to be associated with increased oxidative stress and alterations in antioxidant status. The findings also suggest that lifestyle and dietary antioxidant intake may play a contributory role in modulating these effects.

## 1 Background

Infertility among males is a multifactorial disorder that is known to be one of the major causes of nearly half of all infertile couples around the world (1). Varicocele is one of the most commonly recognized disorders contributing to the cause of infertility among males, with an incidence rate of about 15–20 percent among normal individuals and 40 percent among infertile patients (2). Varicocele is associated with pathological dilation and tortuosity of the pampiniform plexus, leading to disturbances such as impaired regulation of testicular temperature, hypoxia, and microcirculatory disruption, thereby impairing sperm production (3). There is emerging evidence that a reduction in reproductive potential in males could be due, in part, to several interrelated factors (4); however, there is a growing consensus among researchers that oxidative stress, defined as an imbalance resulting from excessive quantities of reactive oxygen species and diminished antioxidant defenses, plays a significant role (5). Pathological conditions associated with varicocele lead to significantly increased ROS generation within the testicles and seminal plasma, ultimately resulting in spermatozoal membrane lipid peroxidation, mitochondrial dysfunction, and damage to spermatozoal genetic material (6).

An increasing number of studies have shown that oxidative stress is an essential player in the pathophysiology of male infertility (7). It describes a condition in which ROS generation exceeds the body’s capacity to counteract their damaging effects through antioxidant defenses, leading to various forms of harm at the cellular and molecular levels, especially in spermatozoa (8). Spermatozoa are particularly susceptible to oxidative stress due to their low capacity to cope with it and their abundance of polyunsaturated fatty acids (9).

A condition referred to as male oxidative stress infertility (MOSI) has been suggested to describe a form of male infertility where oxidative stress plays the key role in its onset (10). Male oxidative stress infertility describes a clinical situation in which infertility arises due to the accumulation of ROS either through increased production or a reduction in antioxidant capabilities in the absence of other major causes of infertility (9). Thus, oxidative stress should be considered the main cause of male infertility in such cases rather than just one of many factors (10–12).

Dietary antioxidants and micronutrients, including vitamins, trace elements, amino acids, and bioactive compounds, have been extensively examined for their ability to neutralize or prevent the effects of ROS and maintain redox homeostasis in human physiological systems (13). The primary mechanism of action is either by directly scavenging free radicals or by acting as cofactors for endogenous antioxidant enzymes to reduce oxidative damage at the cellular level (14). Several clinical interventional studies across the reproductive sciences have demonstrated that the supplementation of dietary antioxidants or micronutrients has resulted in significant improvements in semen quality and reductions in SDF in the sub-fertile male populations analyzed, suggesting that the increase in the systemic availability of antioxidants may help to reduce the effects of oxidative injury mediated by ROS (15).

The mechanistic relationship of varicocele, oxidative stress, and deterioration of semen quality is complex. The rise in local scrotal temperature and blood stasis in varicocele results in hypoxia and increased ROS production. The direct targets of excess ROS are sperm plasma membranes, which are highly enriched in polyunsaturated fatty acids, leading to lipid peroxidation, loss of membrane integrity, and reduced motility (16, 17). Antioxidants in the diet can restore this deficiency, stabilize claims in mitochondria, and prevent the oxidation of sperm DNA. However, their mechanism of action and dose–response depend on the compound. As an illustration, micronutrient antioxidants, including folic acid and zinc, have been shown to reduce seminal ROS levels and improve implantation rates in couples undergoing fertility treatment relative to their matched controls (18, 19). The objective of this study is to analyze the effects of dietary antioxidants and micronutrient status on oxidative stress in patients with varicocele and its effects on semen quality.

## 2 Construction and content

### 2.1 Study design and participants

**Figure 1:**
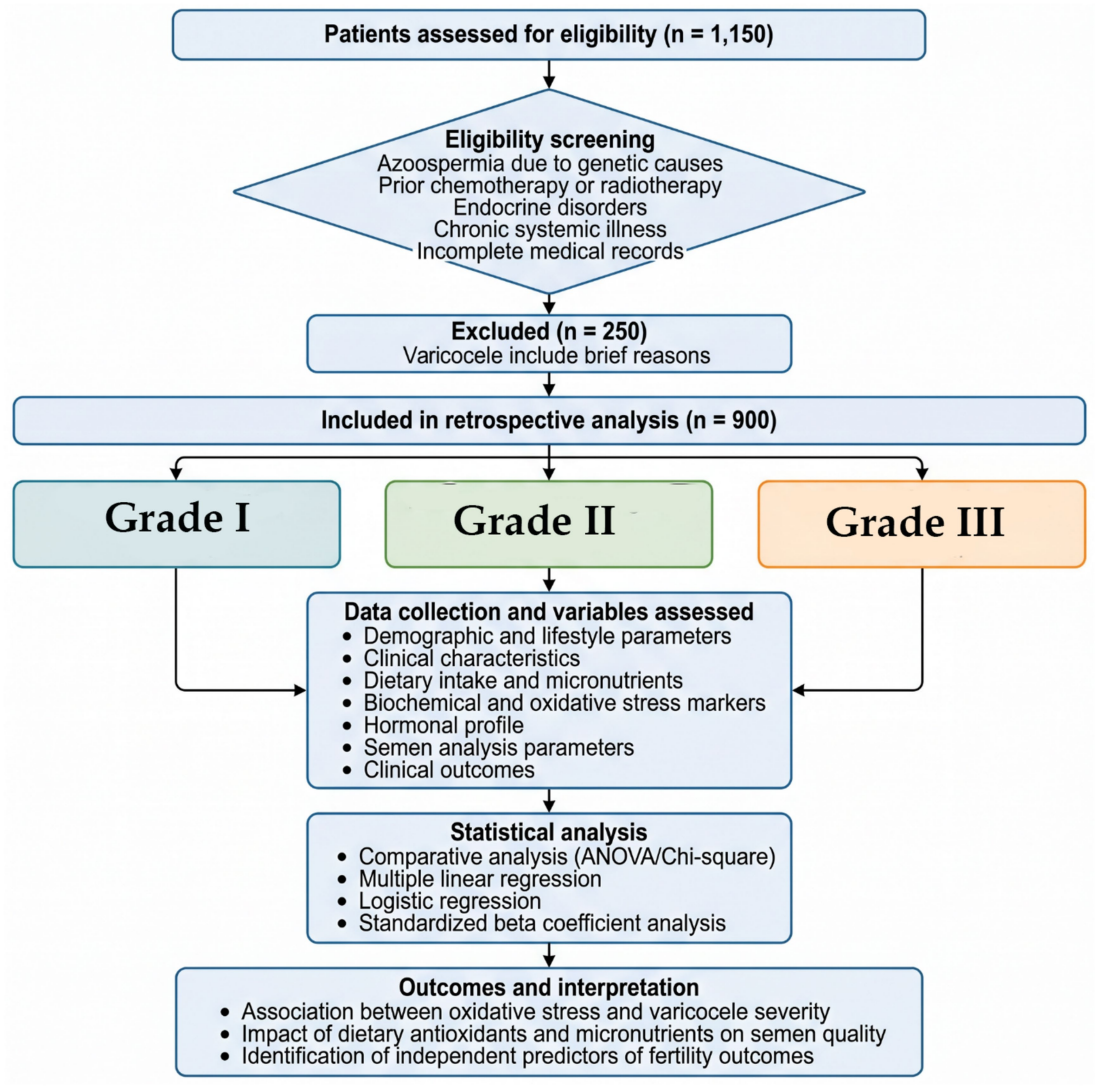
Flow chart of study design.

The retrospective cohort study took place from 2021 to 2023. Researchers examined medical records of 900 men aged 20–45 who had both a clinical and a Doppler-confirmed diagnosis of varicocele. The study assessed varicocele severity using physical examination and Doppler ultrasonography, yielding standard criteria defining three severity levels (Grade I: 300 cases, Grade II: 400 cases, Grade III: 200 cases) (Figure 1).

### 2.2 Inclusion and exclusion criteria

#### 2.2.1 Inclusion criteria

Men aged 20–45 years with varicocele and infertility (≥12 months of unprotected intercourse). The study required complete medical records, including demographic information and lifestyle details, as well as anthropometric measurements, dietary data, biochemical results, hormonal levels, and semen analysis results. The study required at least one semen analysis, which followed the WHO 2021 guidelines. The study required available reproductive outcomes, which included spontaneous conception and ART use. The research examined patients who received evaluation and treatment between 2021 and 2023.

#### 2.2.2 Exclusion criteria

Genetically proven cases of male infertility caused by conditions such as Klinefelter syndrome or Y-chromosome microdeletion syndromes were not included in the study. Men with obstructive azoospermia or congenital absence of the vas deferens were excluded from the study. Moreover, men with systemic diseases associated with impaired fertility, like uncontrolled diabetes mellitus, chronic renal dysfunction, and chronic liver dysfunction, as well as patients with a past medical history of cancer or injury to the urogenital tract, were not included. In addition, patients with an incomplete clinical record that did not allow evaluation of their semen analysis, hormonal levels, or oxidative stress status were excluded. Men with a history of use of any form of exogenous testosterone or anabolic steroids for at least 6 months before inclusion into the study were not enrolled. Patients with ages outside the range of 20 to 45 years old were not included in the study. When assessing reproduction, couples with diagnosed female infertility were not recruited.

### 2.3 Data collection

Data for the current retrospective analysis were obtained from prior medical and electronic health records. Existing records contained data on patients’ demographics and anthropometrics, such as age, height, weight, body mass index (BMI), and waist and hip circumferences. Additionally, data on certain lifestyle factors like smoking, alcohol intake, level of physical activity, occupation, and medical history were obtained from existing clinical records. Moreover, dietary assessment data collected earlier using a valid food frequency questionnaire were obtained from previous records and included information on energy, nutrient, and antioxidant intake over the past month.

### 2.4 Semen analysis and DNA integrity

In the current retrospective study, the data were retrieved from laboratory records that belonged to participants who previously submitted their semen samples after being advised to abstain for 3–5 days. The semen analysis was conducted according to the WHO 2021 criteria (20), which included examination of the sample volume, sperm concentration, motility, morphology, and viability. Additionally, other recorded data included sperm DNA fragmentation via the terminal deoxynucleotidyl transferase-mediated dUTP nick-end labeling test and chromatin maturation via aniline blue staining.

### 2.5 Biochemical and hormonal assessments

In this retrospective study, data were obtained from the electronic medical records of participants who had undergone a clinical examination. The previous blood samples obtained during regular health check-ups were used to obtain information on biochemical and hormone levels. Data included serum levels of micronutrients (Zn, Se, Cu), reproductive hormones (T, FSH, LH), glucose and insulin, and the Homeostasis Model Assessment of Insulin Resistance (HOMA-IR). Oxidative stress biomarkers, including malondialdehyde (MDA), superoxide dismutase (SOD), and total antioxidant status, were extracted from the available laboratory test results.

#### 2.5.1 Treatment and clinical management approach

Since this was a retrospective observational study, treatment was provided by clinicians according to usual practice guidelines, rather than protocol-based intervention assignments. Patients were treated on the basis of their varicocele status, results from sperm analysis tests, period of infertility, hormonal status, and reproductive intentions.

The patient medical records were examined to determine which individuals received conservative treatment, antioxidant treatments, varicocelectomy procedures, assisted reproductive technology, or any combination thereof. In general, varicocelectomy was most often done for patients who had Grade II to Grade III disease and especially those that had abnormalities in their semen, were infertile, had testicular pain, or showed signs of atrophy of their testicles. The surgical treatment involved microsurgical subinguinal or inguinal varicocelectomy procedure.

Patients who had no surgical intervention were managed with a conservative approach such as lifestyle modification, fertility surveillance, and medical therapy. Antioxidant supplements were prescribed to some patients by their doctors at their discretion and included vitamin C, vitamin E, zinc, selenium, Coenzyme Q10, and mixed antioxidants. The use of antioxidants was captured from prescription and follow-up reports.

IVF/ICSI procedures were performed on couples that were found to have infertility issues, abnormal semen analysis, advanced age of the female partner, and those who failed to conceive spontaneously despite having treatment. Clinical variables such as postoperative improvement in semen quality, decrease in oxidative stress, pregnancy following varicocelectomy, ART cycles, and birth of live babies were obtained from follow-up information.

In analyzing the data, variables related to treatment such as varicocelectomy, use of antioxidants, number of ART cycles, postoperative improvement of semen quality, and pregnancy rates were considered among the clinical outcome variables. However, since there was no randomization of treatment assignment, causality could not be claimed.

Accordingly, only approximately 30% of men in the Grade I varicocele subgroup met these criteria and therefore underwent surgical management. The remaining patients were managed conservatively in accordance with standard practice guidelines. Conservative management included regular clinical and ultrasonographic follow-up, lifestyle modification (e.g., avoidance of heat exposure and prolonged standing), and antioxidant supplementation aimed at reducing oxidative stress and improving semen quality.

### 2.6 Clinical outcomes

ART involves medical procedures that enable an individual or couple to conceive through the manipulation of both egg cells and sperm. Examples of ART include *in vitro* fertilization (IVF), intracytoplasmic sperm injection (ICSI), and embryo transfer. The researchers obtained data about varicocelectomy surgical procedures and all ART procedures from previously medical records of the patients, including IVF and ICSI, and all cases of natural conception, live birth, miscarriage, and the period of patient monitoring. The clinic reports were used to confirm the outcomes of ART procedures.

### 2.7 Statistical analysis

The study reported continuous variables as mean ± standard deviation, whereas categorical variables were reported as percentages. The study compared different varicocele severity groups using ANOVA and chi-square tests. Multiple linear regression identified factors that predicted sperm concentration, motility, and DNA fragmentation, while logistic regression identified factors that predicted spontaneous conception. Researchers used standardized beta coefficients to measure the extent to which different variables affected outcomes. The researchers determined statistical significance at *p* < 0.05. The researchers used SPSS Version 26.0 for their analyses, a statistical software package developed by IBM Corp in Armonk, New York, USA.

### 2.8 Ethical considerations

This research was approved by the Ethics Committee of Nanjing Jinling Hospital (Approval Number: LC20250911). The Institutional Review Board approved the study. The researchers used only de-identified patient records. This was because the study required reviewing past data, and informed consent wasn’t necessary.

## 3 Results

### 3.1 Demographic and lifestyle profiles across varicocele severity groups

There were a total of 900 male participants, stratified into three groups of 300 each in the Grade I group, 400 in the Grade II group, and 200 in the Grade III group. Age averaged 32.4 ± 5.8 years, with no significant differences among groups (*p* = 0.12). Also, BMI, WC, HC, and WHR were similar across groups (*p* > 0.05). The lifestyle parameters, which included smoking (28%), alcohol consumption (12%), occupational sedentariness (40%), and exercise frequency, did not differ significantly among the groups. However, the number of sleeping hours was significantly lower in the Grade III category (6.5 ± 1.3 h) than in the Grade I group (7.0 ± 1.1 h, *p* = 0.02). The self-reported stress score was significantly higher in the Grade III group (6 ± 2) than in the Grade I group (4 ± 2, *p* = 0.01). Regarding clinical parameters, varicocele laterality varied significantly among the three groups (*p* = 0.04), with the number of bilateral varicoceles increasing with greater severity. In addition, testicular volume decreased significantly with increased disease severity (*p* = 0.01). Furthermore, there was a significant difference in the varicocele grade distribution (*p* = 0.001) (Table 1).

**Table 1:** Demographics, anthropometrics, and lifestyle.

Parameter	Total (*n* = 900)	Grade I (*n* = 300)	Grade II (*n* = 400)	Grade III (*n* = 200)	*p* value
Age (years)	32.4 ± 5.8	31.8 ± 5.5	32.5 ± 6.0	33.2 ± 5.9	0.12
BMI (kg/m^2^)	25.8 ± 3.7	25.5 ± 3.6	25.9 ± 3.8	26.0 ± 3.9	0.45
Waist circumference (cm)	88 ± 10	87 ± 9	89 ± 11	90 ± 12	0.08
Hip circumference (cm)	98 ± 8	97 ± 7	99 ± 8	100 ± 9	0.09
Waist-to-hip ratio	0.90 ± 0.05	0.90 ± 0.04	0.90 ± 0.05	0.91 ± 0.05	0.12
Smoking (%)	28%	25%	30%	31%	0.22
Alcohol use (%)	12%	10%	13%	15%	0.18
Physical activity (MET-min/week)	1,200 ± 400	1,300 ± 390	1,180 ± 420	1,150 ± 410	0.07
Sedentary occupation (%)	40%	38%	42%	41%	0.55
Sleep duration (hours/night)	6.8 ± 1.2	7.0 ± 1.1	6.7 ± 1.3	6.5 ± 1.3	0.02
Stress level (0–10)	5 ± 2	4 ± 2	5 ± 2	6 ± 2	0.01
Socioeconomic status (low/med/high)	20/55/25%	18/57/25%	21/54/25%	22/53/25%	0.30
Marital status (married %)	70%	68%	72%	71%	0.50
Family history of infertility (%)	10%	9%	11%	10%	0.87
Comorbidities (%)	18%	15%	19%	20%	0.34
Varicocele laterality (uni/bilateral %)	60/40	65/35	58/42	55/45	0.04
Testicular volume (mL)	18.5 ± 3.0	19.2 ± 2.9	18.1 ± 3.1	17.8 ± 3.2	0.01
History of urogenital infection (%)	15%	12%	16%	18%	0.08
Previous scrotal surgery (%)	5%	4%	5%	6%	0.60
Duration of infertility (years)	3.2 ± 1.5	2.9 ± 1.4	3.3 ± 1.6	3.5 ± 1.7	0.08
Medication use (%)	20%	18%	21%	22%	0.45
Heat exposure (%)	25%	22%	26%	27%	0.18
Occupation-related toxin exposure (%)	8%	6%	8%	10%	0.09
BMI category (normal/overweight/obese)	40/45/15%	42/44/14%	40/46/14%	38/45/17%	0.33
Varicocele grade (I/II/III)	30/50/20%	100/120/80	200/150/50	0/130/70	0.001

### 3.2 Dietary intake, micronutrient status, and semen quality across varicocele severity

Analysis of diet revealed statistically significant decreases in protein (*p* = 0.04), omega-3 fatty acids (*p* = 0.03), and important micronutrients including vitamin A, C, D, and E, zinc, selenium, magnesium, and iron. The most Grade III ill subjects consistently consumed less than the mildly affected individuals. Fruit and vegetable consumption was statistically significantly lower among the most Grade III ill individuals (*p* = 0.01). The results of the biochemical assessment supported those from the dietary evaluation. Serum concentrations of zinc, selenium, magnesium, iron, and antioxidants were significantly lower in Grade III cases (all *p* < 0.05). Biomarkers of oxidative stress, such as malondialdehyde and ROS, showed significant elevation with increasing severity (*p* = 0.01 and *p* = 0.02, respectively). Antioxidants such as total antioxidant capacity (TAC), reduced glutathione (GSH), and SOD showed significantly lower concentrations (*p* < 0.05). Regarding the hormone profile, testosterone showed a significant decrease, while FSH increased significantly with increasing severity (*p* ≤ 0.05), indicating poor spermatogenesis. C-reactive protein (CRP), an inflammatory biomarker, showed significant elevation in Grade III cases (*p* = 0.04). Findings on semen parameters showed a continuous decline in sperm concentration, motility, morphology, and vitality across the severity groups (*p* ≤ 0.03 for all parameters). There was higher sperm DNA fragmentation and apoptosis (*p* = 0.01), together with increased seminal oxidative stress and reduced antioxidant levels (Table 2).

**Table 2:** Dietary intake and micronutrients.

Parameter	Grade I (*n* = 300)	Grade II (*n* = 400)	Grade III (*n* = 200)	*p* value
Dietary intake and micronutrients				
Energy intake (kcal/day)	2,250 ± 420	2,180 ± 390	2,150 ± 410	0.15
Protein (g/day)	88 ± 18	84 ± 21	80 ± 22	0.04
Carbohydrates (g/day)	290 ± 45	275 ± 50	270 ± 55	0.05
Fat (g/day)	78 ± 14	74 ± 16	72 ± 16	0.06
Omega-3 (g/day)	1.6 ± 0.5	1.5 ± 0.4	1.4 ± 0.5	0.03
Omega-6 (g/day)	10 ± 2	10 ± 3	9 ± 3	0.12
Vitamin A (μg/day)	850 ± 180	780 ± 210	750 ± 220	0.04
Vitamin C (mg/day)	85 ± 22	78 ± 24	74 ± 26	0.03
Vitamin E (mg/day)	13 ± 3	12 ± 4	11 ± 5	0.05
Vitamin D (ng/mL)	27 ± 7	24 ± 8	22 ± 9	0.01
Folate (μg/day)	370 ± 90	340 ± 105	330 ± 110	0.06
Zinc (mg/day)	13 ± 3	12 ± 3	11 ± 4	0.02
Selenium (μg/day)	75 ± 18	68 ± 21	65 ± 22	0.03
Copper (μg/day)	1.3 ± 0.3	1.2 ± 0.4	1.1 ± 0.4	0.05
Magnesium (mg/day)	370 ± 75	340 ± 85	330 ± 90	0.04
Iron (mg/day)	13 ± 2	12 ± 3	11 ± 3	0.03
Beta-carotene (μg/day)	3,700 ± 1,100	3,400 ± 1,300	3,300 ± 1,250	0.04
Lycopene (μg/day)	6,200 ± 1,400	5,900 ± 1,600	5,800 ± 1,500	0.05
Polyphenols (mg/day)	420 ± 110	390 ± 125	380 ± 130	0.06
Flavonoids (mg/day)	160 ± 45	145 ± 50	140 ± 55	0.04
Fiber (g/day)	27 ± 7	24 ± 8	23 ± 9	0.03
Water intake (L/day)	2.1 ± 0.4	2.0 ± 0.5	1.9 ± 0.5	0.08
Fruit and vegetable servings/day	5 ± 1.4	4.4 ± 1.5	4 ± 1.6	0.01
Caffeine intake (mg/day)	115 ± 55	122 ± 60	125 ± 65	0.09
Antioxidant supplement use (%)	18%	14%	12%	0.12
Biochemical and hormonal parameters				
Serum zinc (μg/dL)	100 ± 14	94 ± 15	90 ± 16	0.02
Serum selenium (μg/L)	95 ± 18	88 ± 20	85 ± 22	0.03
Serum copper (μg/dL)	112 ± 14	109 ± 15	108 ± 16	0.05
Serum magnesium (mg/dL)	2.2 ± 0.2	2.1 ± 0.3	2.0 ± 0.3	0.04
Serum iron (μg/dL)	88 ± 22	84 ± 26	80 ± 28	0.03
Total antioxidant capacity (TAC, mmol/L)	1.5 ± 0.3	1.4 ± 0.3	1.3 ± 0.3	0.04
Malondialdehyde (MDA, μmol/L)	2.8 ± 0.7	3.3 ± 0.8	3.6 ± 0.9	0.01
Reactive oxygen species (ROS, RLU/s/million sperm)	100 ± 35	125 ± 38	135 ± 42	0.02
Glutathione (GSH, μmol/L)	6.5 ± 1.3	5.9 ± 1.4	5.5 ± 1.6	0.02
Superoxide dismutase (SOD, U/mL)	2.8 ± 0.6	2.4 ± 0.7	2.2 ± 0.8	0.01
Catalase activity (U/mL)	48 ± 9	44 ± 10	42 ± 11	0.03
Testosterone (ng/dL)	500 ± 115	475 ± 120	460 ± 125	0.05
FSH (mIU/mL)	6.2 ± 2.3	7.0 ± 2.5	7.5 ± 2.6	0.03
LH (mIU/mL)	5.2 ± 1.7	5.6 ± 1.8	5.8 ± 1.9	0.08
Estradiol (pg/mL)	27 ± 7	28 ± 8	29 ± 9	0.21
Prolactin (ng/mL)	11 ± 4	12 ± 5	13 ± 5	0.12
T/E ratio	18 ± 4	16 ± 5	15 ± 6	0.02
Serum albumin (g/dL)	4.3 ± 0.3	4.2 ± 0.3	4.1 ± 0.3	0.04
Serum total protein (g/dL)	7.3 ± 0.5	7.2 ± 0.6	7.1 ± 0.6	0.06
Blood glucose (mg/dL)	94 ± 11	96 ± 12	96 ± 13	0.12
HbA1c (%)	5.3 ± 0.4	5.5 ± 0.5	5.5 ± 0.6	0.08
Serum vitamin C (mg/dL)	0.9 ± 0.2	0.8 ± 0.2	0.7 ± 0.2	0.03
Serum vitamin E (mg/dL)	1.3 ± 0.3	1.2 ± 0.3	1.1 ± 0.3	0.04
Serum folate (ng/mL)	13 ± 3	12 ± 3	11 ± 3	0.05
Serum homocysteine (μmol/L)	11 ± 3	12 ± 4	13 ± 5	0.02
C-reactive protein (mg/L)	3.2 ± 1.1	3.6 ± 1.2	3.8 ± 1.3	0.04
Semen parameters and DNA integrity				
Semen volume (mL)	3.0 ± 0.8	2.7 ± 0.9	2.6 ± 1.0	0.02
Sperm concentration (million/mL)	50 ± 14	40 ± 15	35 ± 16	0.01
Total motility (%)	60 ± 11	53 ± 12	50 ± 13	0.01
Progressive motility (%)	40 ± 9	34 ± 10	30 ± 11	0.01
Normal morphology (%)	7 ± 3	6 ± 3	5 ± 2	0.03
Vitality (%)	75 ± 9	69 ± 10	65 ± 11	0.01
Sperm DNA fragmentation (%)	20 ± 8	26 ± 9	30 ± 11	0.01
Sperm chromatin maturity (%)	65 ± 14	58 ± 15	55 ± 16	0.02
Seminal ROS	100 ± 35	125 ± 38	135 ± 42	0.02
Seminal TAC	1.5 ± 0.3	1.4 ± 0.3	1.3 ± 0.3	0.04
Seminal zinc (μg/dL)	130 ± 22	118 ± 26	115 ± 28	0.03
Seminal selenium (μg/L)	105 ± 18	98 ± 20	95 ± 22	0.04
Seminal vitamin C (mg/dL)	1.3 ± 0.3	1.2 ± 0.3	1.1 ± 0.3	0.03
Seminal vitamin E (mg/dL)	0.9 ± 0.2	0.8 ± 0.2	0.7 ± 0.2	0.04
pH	7.5 ± 0.3	7.5 ± 0.3	7.4 ± 0.3	0.12
Liquefaction time (min)	24 ± 7	25 ± 8	26 ± 9	0.08
Viscosity (scale 0–3)	1 ± 0.4	1 ± 0.5	1 ± 0.6	0.14
Sperm aggregation (%)	8 ± 4	11 ± 5	12 ± 6	0.05
Sperm adhesion (%)	12 ± 5	16 ± 6	18 ± 7	0.04
Sperm apoptosis (%)	15 ± 7	21 ± 8	25 ± 9	0.01
Round cells (million/mL)	2.0 ± 1.0	2.6 ± 1.3	2.8 ± 1.4	0.03
Leukocytes (million/mL)	0.4 ± 0.2	0.5 ± 0.3	0.6 ± 0.3	0.04
Seminal osmolarity (mOsm/kg)	295 ± 18	302 ± 22	305 ± 24	0.05
Round cell apoptosis (%)	10 ± 4	13 ± 5	15 ± 6	0.02

### 3.3 Oxidative stress markers and antioxidant enzyme status in varicocele-associated infertility

**Figure 2:**
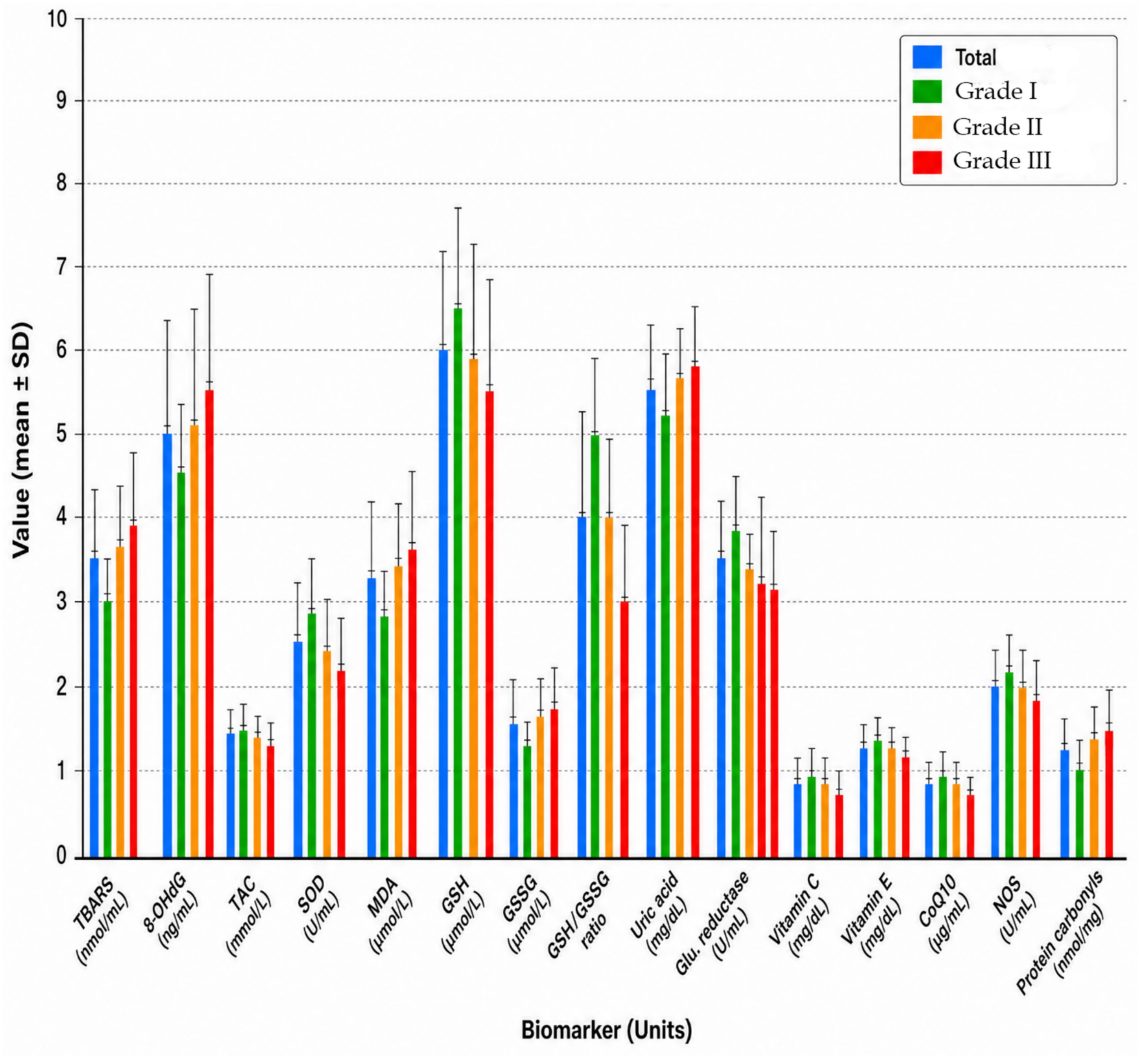
Oxidative stress biomarkers by disease severity.

The signs of oxidative stress consistently increased with disease severity. The level of malondialdehyde (MDA), reactive oxygen species (ROS), lipid peroxidation (TBARS), protein carbonyls, and oxidative DNA damage (8-hydroxydeoxyguanosine - 8-OHdG) significantly increased in the Grade III group (*p* ≤ 0.02). The level of nitric oxide decreased. There was evidence of reduced antioxidant enzyme activity. Levels of superoxide dismutase (SOD), catalase, glutathione peroxidase (GPx), and glutathione reductase were significantly lower in the Grade III cases (*p* < 0.05). The ratio of glutathione (GSH)/glutathione disulfide (GSSG) was significantly lower (*p* = 0.01). Levels of micronutrients such as zinc, selenium, vitamin C, vitamin E, and coenzyme Q10 were also significantly lower (*p* < 0.05) with increasing severity. The total scavenging activity of ROS was lower (Table 3, Figure 2).

**Table 3:** Oxidative stress and antioxidant enzymes.

Parameter	Grade I (*n* = 300)	Grade II (*n* = 400)	Grade III (*n* = 200)	*p* value
Reactive oxygen species (ROS, RLU/s/million sperm)	100 ± 35	125 ± 38	135 ± 42	0.02
Catalase (U/mL)	48 ± 9	44 ± 10	42 ± 11	0.03
Glutathione peroxidase (GPx, U/mL)	37 ± 7	34 ± 8	32 ± 9	0.02
Nitric oxide (NO, μmol/L)	38 ± 9	34 ± 10	32 ± 11	0.04
Advanced oxidation protein products (AOPP, μmol/L)	75 ± 18	82 ± 21	85 ± 23	0.03
Selenium (plasma, μg/L)	95 ± 18	88 ± 20	85 ± 22	0.03
Zinc (plasma, μg/dL)	100 ± 14	94 ± 15	90 ± 16	0.02
Lipid hydroperoxides (μmol/L)	110 ± 28	125 ± 32	130 ± 35	0.02
Total ROS scavenging (%)	78 ± 9	74 ± 10	72 ± 11	0.03

### 3.4 Reproductive hormones and endocrine profile across varicocele severity

**Figure 3:**
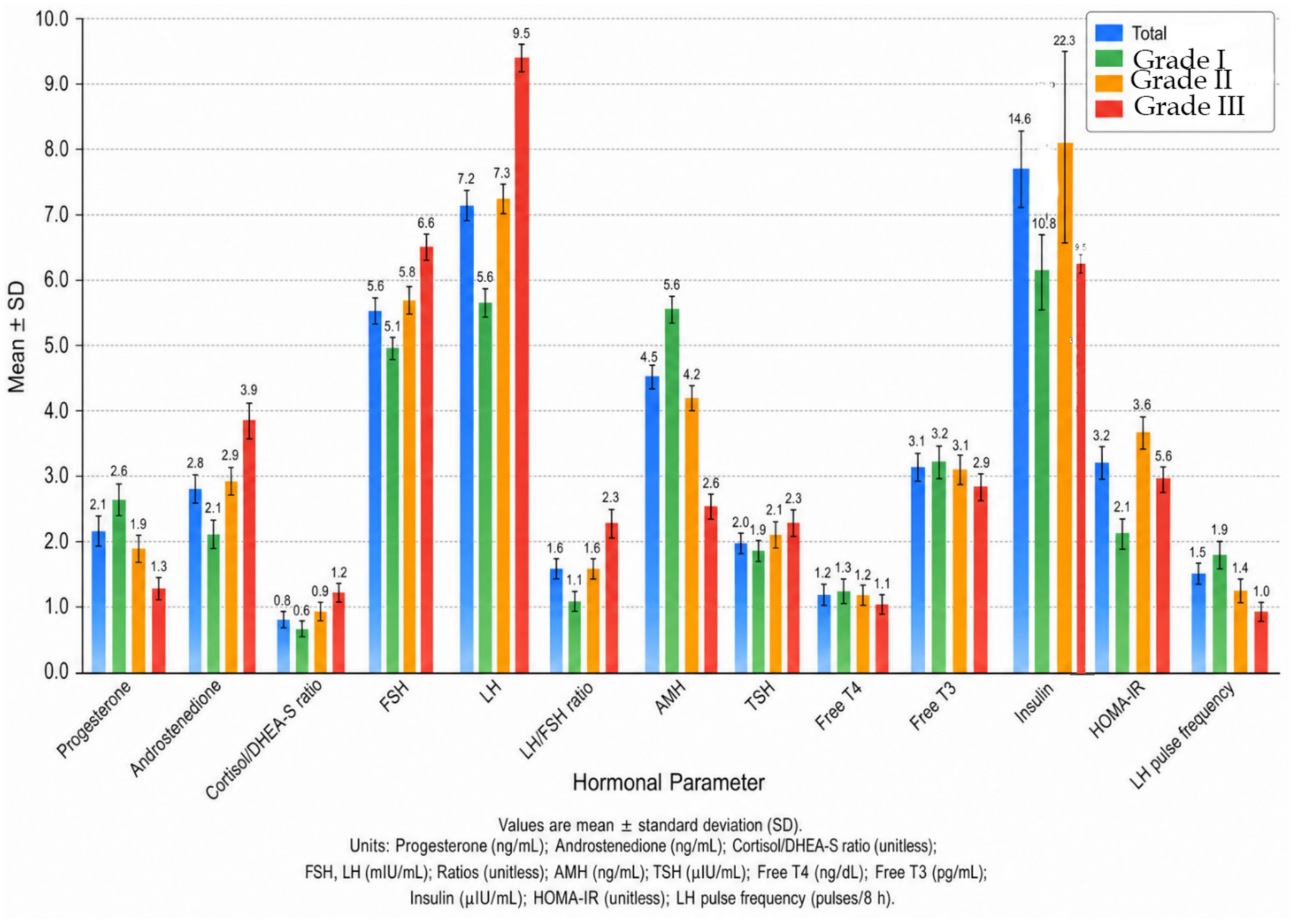
Hormonal parameters by severity group.

The endocrine assessment revealed notable changes between groups. Both total and free testosterone showed marked reductions in the Grade III group (*p* = 0.05 and *p* = 0.03, respectively). The FSH levels were noted to increase significantly as severity rose (*p* = 0.03). Marked decreases in sex hormone-binding globulin (SHBG), testosterone-to-LH ratio, anti-Müllerian hormone (AMH), inhibin B, and DHEA-S concentrations were noted in Grade III cases (*p* < 0.05). The insulin resistance index (HOMA-IR) was significantly higher as severity increased (*p* = 0.03). There were no marked differences in LH, estradiol, prolactin, thyroid hormone, or progesterone concentrations. However, the LH pulse frequency was significantly reduced in Grade III cases (*p* = 0.04) (Table 4, Figure 3).

**Table 4:** Reproductive Hormones and Endocrine Profile.

Parameter	Total (*n* = 900)	Grade I (*n* = 300)	Grade II (*n* = 400)	Grade III (*n* = 200)	*p* value
Testosterone (ng/dL)	480 ± 120	500 ± 115	475 ± 120	460 ± 125	0.05
Free testosterone (ng/dL)	12 ± 3	13 ± 2	12 ± 3	11 ± 3	0.03
Estradiol (pg/mL)	28 ± 8	27 ± 7	28 ± 8	29 ± 9	0.21
Prolactin (ng/mL)	12 ± 5	11 ± 4	12 ± 5	13 ± 5	0.12
SHBG (nmol/L)	40 ± 10	42 ± 9	39 ± 10	38 ± 11	0.04
Testosterone/LH ratio	90 ± 25	95 ± 22	88 ± 25	85 ± 27	0.03
Inhibin B (pg/mL)	120 ± 40	130 ± 35	115 ± 40	110 ± 45	0.02
DHEA-S (μg/dL)	300 ± 100	320 ± 90	295 ± 100	280 ± 110	0.04
Cortisol (μg/dL)	14 ± 5	13 ± 4	14 ± 5	15 ± 6	0.08
Estradiol/testosterone ratio	0.06 ± 0.02	0.05 ± 0.02	0.06 ± 0.02	0.06 ± 0.03	0.07
Estrone (pg/mL)	50 ± 12	48 ± 10	51 ± 12	52 ± 14	0.06

### 3.5 Clinical outcomes, assisted reproductive technology use, and pregnancy rates in varicocele-associated infertility

**Figure 4:**
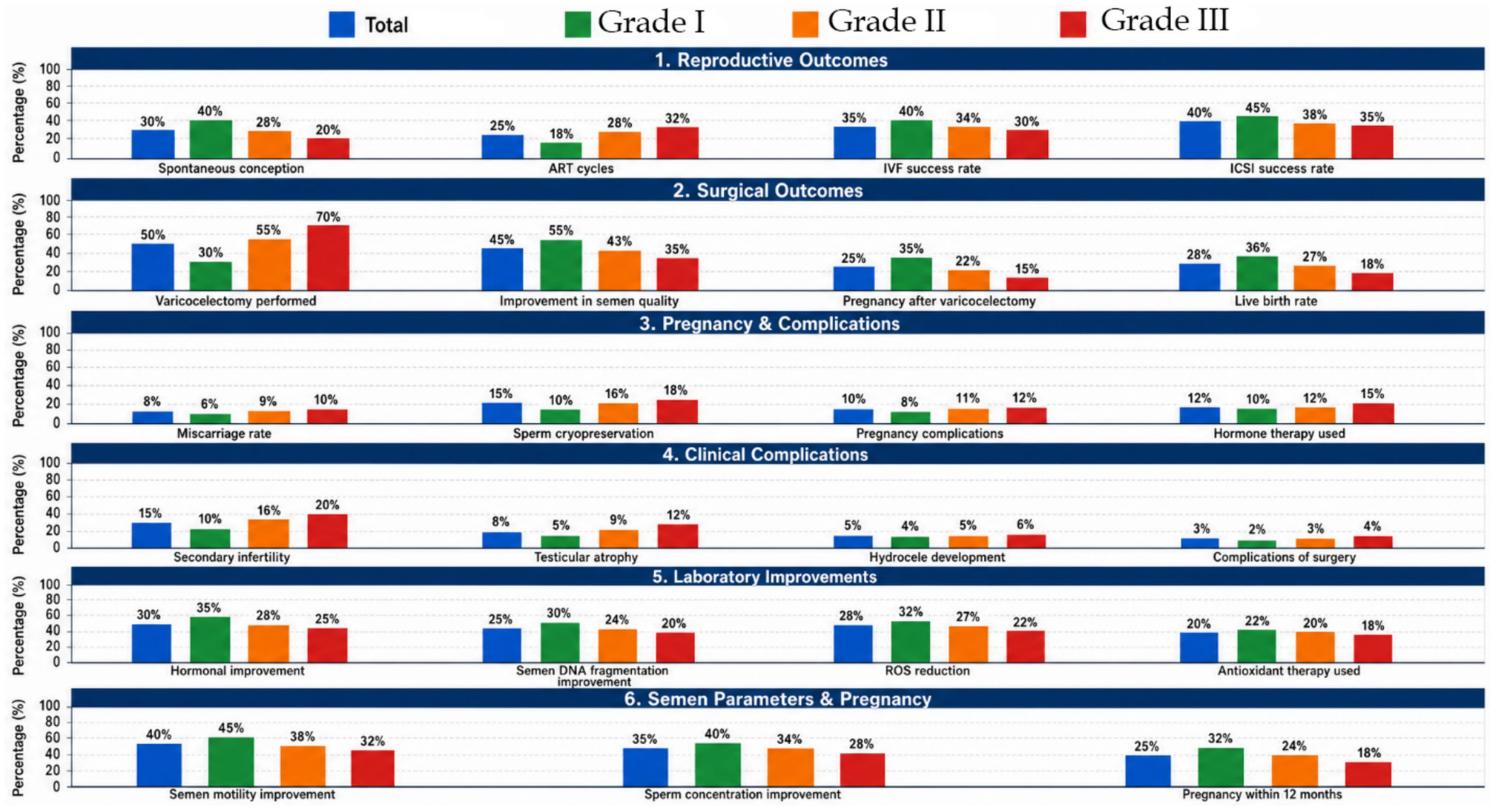
Clinical outcomes by varicocele severity.

There were significant differences in clinical outcomes between groups. The spontaneous conception rate decreased from 40% in Grade I disease to 20% in Grade III disease (*p* < 0.001). On the other hand, ART use increased with the severity (*p* = 0.002), with more cycles needed in Grade III cases (*p* = 0.03). IVF/ICSI conception rates were significantly higher in Grade I disease (*p* ≤ 0.05). There was an increase in time to conception with increasing severity (*p* = 0.03). Varicocelectomies were performed more often in Grade III cases (70%) than in Grade I cases (30%) (*p* < 0.001). The postoperative results indicated significant improvements in semen quality and pregnancy rates in Grade I cases (*p* ≤ 0.01). The birth rate was also significantly lower in Grade III cases (18% vs. 36% in Grade I cases; *p* < 0.001). Moreover, secondary infertility and testicular atrophy were observed more commonly among Grade III cases (*p* ≤ 0.02). There were significant improvements in sperm motility, sperm concentration, sperm DNA fragmentation index, and ROS in Grade I cases (*p* < 0.05) (Table 5, Figure 4).

**Table 5:** Clinical outcomes, ART use, and pregnancy rates.

Parameter	Grade I (*n* = 300)	Grade II (*n* = 400)	Grade III (*n* = 200)	*p* value
ART cycles per patient	1.6 ± 0.5	1.9 ± 0.6	2.0 ± 0.7	0.03
Assisted fertilization type (%)	45/40/15	40/45/15	44/38/18	
Follow-up duration (months)	24 ± 5	25 ± 6	23 ± 7	0.20
Time to conception (months)	10 ± 5	12 ± 6	14 ± 7	0.03

### 3.6 Multiple linear regression analysis predicting sperm concentration in varicocele-associated infertility

Multiple linear regression analysis revealed various independent predictors for sperm concentration. Increased varicocele severity (*β* = −0.25, *p* < 0.001), increased levels of ROS (*β* = −0.20, *p* < 0.001) and increased MDA levels (*β* = −0.15, *p* < 0.001) were important negative predictors. Other negative predictors included BMI (*p* ≤ 0.004) and smoking (p ≤ 0.004). On the other hand, dietary antioxidants such as zinc (*β* = 0.18, *p* < 0.001), selenium (*p* = 0.008), vitamin C (*p* = 0.003), and vitamin E (*p* = 0.004) were important positive predictors (Table 6).

**Table 6:** Multiple linear regression predicting sperm concentration.

Predictor	*B* (unstandardized)	SE	Beta (standardized)	*t*	*p* value
Age (years)	−0.25	0.08	−0.12	−3.12	0.002
BMI (kg/m^2^)	−0.35	0.10	−0.15	−3.50	<0.001
Varicocele grade (I–III)	−4.0	0.8	−0.25	−5.00	<0.001
Smoking (yes = 1)	−3.5	1.2	−0.10	−2.92	0.004
Vitamin C (mg/day)	0.15	0.05	0.12	3.00	0.003
Vitamin E (mg/day)	0.20	0.07	0.10	2.86	0.004
Zinc (mg/day)	0.50	0.12	0.18	4.17	<0.001
Selenium (μg/day)	0.40	0.15	0.09	2.67	0.008
ROS (RLU/s/10^6^ sperm)	−0.08	0.02	−0.20	−4.00	<0.001
MDA (μmol/L)	−2.0	0.5	−0.15	−4.00	<0.001
Constant	60	5	–	12	<0.001

### 3.7 Multiple linear regression analysis predicting sperm motility in varicocele-associated infertility

Likewise, the factors influencing sperm motility exhibited similar tendencies. The level of varicocele grading (*β* = −0.22, *p* < 0.001) and the biomarkers of oxidative stress (ROS and MDA) acted as negative predictors. Similarly, age, body mass index (BMI), and smoking had a negative impact on motility (*p* ≤ 0.012). On the other hand, antioxidant consumption, especially zinc, vitamin C, and vitamin E, positively affected sperm motility (all *p* ≤ 0.003), emphasizing the protective effects of antioxidants (Table 7).

**Table 7:** Multiple linear regression predicting sperm motility (%).

Predictor	*B*	SE	Beta	*t*	*p* value
Age	−0.20	0.07	−0.10	−2.86	0.004
BMI	−0.30	0.09	−0.12	−3.33	0.001
Varicocele grade	−3.5	0.7	−0.22	−5.00	<0.001
Smoking	−2.5	1.0	−0.08	−2.50	0.012
Vitamin C	0.12	0.04	0.10	3.00	0.003
Vitamin E	0.18	0.06	0.11	3.00	0.003
Zinc	0.40	0.10	0.16	4.00	<0.001
ROS	−0.05	0.02	−0.14	−3.50	<0.001
MDA	−1.5	0.4	−0.12	−3.75	<0.001
Constant	65	5	–	13	<0.001

### 3.8 Logistic regression predicting spontaneous conception in men with varicocele-associated infertility

Results from logistic regression showed that advancing age (OR = 0.95, *p* = 0.02), increased BMI (OR = 0.92, *p* = 0.002), increased varicocele grade (OR = 0.64, *p* < 0.001), and smoking habits (OR = 0.70, *p* = 0.01) were associated with a decreased likelihood of natural conception. On the other hand, increased levels of vitamin C (OR = 1.05), vitamin E (OR = 1.06), and zinc (OR = 1.13) increased chances of conception (*p* ≤ 0.01). Increased levels of ROS and MDA were also associated with decreased odds of conception (Table 8).

**Table 8:** Logistic regression predicting spontaneous conception.

Predictor	*B* (log-odds)	SE	Odds ratio (OR)	95% CI	*p* value
Age	−0.05	0.02	0.95	0.91–0.99	0.02
BMI	−0.08	0.03	0.92	0.87–0.97	0.002
Varicocele grade	−0.45	0.12	0.64	0.51–0.81	<0.001
Smoking	−0.35	0.15	0.70	0.53–0.92	0.01
Vitamin C intake	0.05	0.02	1.05	1.01–1.09	0.01
Vitamin E intake	0.06	0.03	1.06	1.01–1.11	0.01
Zinc intake	0.12	0.04	1.13	1.04–1.22	0.004
ROS	−0.03	0.01	0.97	0.95–0.99	0.003
MDA	−0.10	0.03	0.90	0.84–0.96	0.001

### 3.9 Standardized Beta coefficients for key predictors of semen quality parameters

**Figure 5:**
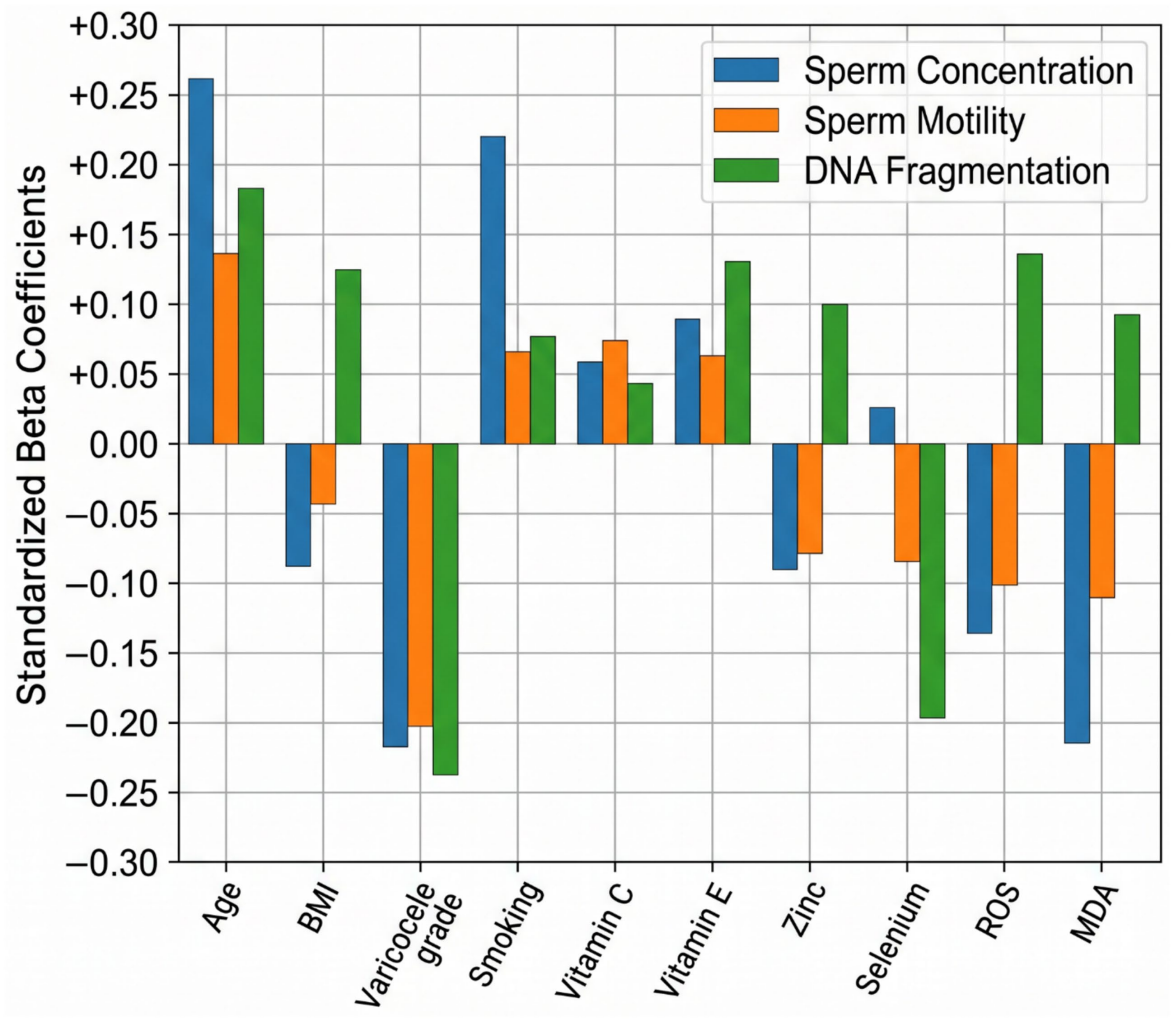
Standardized beta coefficients for predictors of semen quality.

Analysis using standardized beta coefficientss revealed that varicocele grade and oxidative stress markers such as reactive oxygen species (ROS) and malondialdehyde (MDA) were the strongest negative predictors of sperm concentration, motility, and DNA fragmentation. The antioxidants, especially zinc, vitamin C, and vitamin E, had a positive correlation with semen quality variables, whereas age, body mass index (BMI), and cigarette smoking had a Grade II effect. It is important to mention that ROS had the most significant positive relationship with DNA fragmentation (*β* = 0.22) (Figure 5).

## 4 Discussion

### 4.1 Overview of retrospective varicocele infertility observations

In this observational retrospective analysis, we examined 900 cases of infertile patients with varicoceles and showed that there is a significant deterioration in various reproductive, metabolic, and oxidative indices according to their severity level. Our results clearly show that there is a relationship between increased varicocele grade and poor semen quality, high oxidative stress, hormonal imbalances, and decreased pregnancy rate. All of these results are consistent with previous observational analyses regarding this subject matter (21, 22).

Demographic and anthropometric variables, such as age, BMI, and behavioral variables like smoking and drinking alcohol, showed no significant differences between groups depending on varicocele severity. Comparable results have been observed in studies performed using large cohorts of patients, which revealed that severity of varicocele does not correlate with demographic distribution at the initial examination stage (23, 24).

### 4.2 Increased life stress and sleep deprivation increase disease association severity

We noted significantly decreased hours of sleep and increased levels of stress among Grade III varicocele subjects. In previous reports, it was noted that stress and disturbed sleep may contribute to oxidative stress mechanisms and compromise spermatogenesis. In a retrospective study design such as this one, it is not possible to establish causation; however, the results corroborate existing literature on the topic (25, 26).

A decrease in testicular volume was noted along with bilateral varicoceles which increased in frequency in more Grade III patients. As stated in past studies, testicular hypotrophy due to poor venous drainage and increased scrotal temperature has been observed in patients with advanced-stage varicoceles via ultrasound and clinical examination. Our findings further support the theory of structural deterioration presented in past observational research (27, 28).

### 4.3 Malnutrition amplifies pathology of male infertility

Significant decreases in protein, omega-3 fatty acids, and essential micronutrients such as vitamin A, C, D, E, zinc, selenium, and magnesium in more Grade III cases was observed. In the past, research into nutritional epidemiology has shown that malnourishment of antioxidants is common in men with fertility problems. We have expanded on previous work through finding that there exists a correlation between the level of severity and malnutrition (29, 30).

It is shown that there is an increase in ROS, MDA, and TBARS, as well as a reduction in TAC, SOD, GPx, and GSH in patients with Grade III varicocele. It confirms the role of oxidative stress as an important mechanism in varicocele-induced damage to the testes that was revealed in previous studies. The inverse correlation between oxidative stress and antioxidative protection is a strong indicator of oxidative dysbalance suggested by the results of the previous studies (31).

### 4.4 Hormonal disbalance suggests progressive testicular dysfunction

A decrease in testosterone and inhibin B concentrations and an elevation in FSH were revealed. Such changes were demonstrated in previous cross-sectional studies on the issue, which indicated primary testicular dysfunction in advanced varicocele. Low AMH and SHBG suggest damage to Sertoli and Leydig cells as previously described (32).

The decrease in semen parameters such as concentration, motility, morphology, and vitality was significant with an increase in varicocele severity, while an increase was seen in sperm DNA damage and apoptosis. The findings are in line with previously conducted meta-analysis proving that varicocele leads to impaired semen quality along with increased sperm DNA damage. The dose–response effect found in our study reinforces the association of varicocele severity with spermatogenic failure (33).

### 4.5 Oxidative DNA damage causes sperm impairment

Increased levels of DNA damage indicators such as 8-OHdG are significant in patients suffering from Grade III varicocele. Studies conducted earlier using molecular techniques have shown that oxidative stress causes sperm chromatin instability and mitochondrial dysfunction. Our study supports these pathways and implicates oxidative DNA damage as the mediator of impairment (34).

There was an increase in HOMA-IR along with changes in androgen ratios in Grade III varicocele patients, suggesting a role of metabolism in these cases. Earlier research has shown the existence of two-way association between oxidative stress and insulin resistance in male reproductive disorders. The link between metabolic dysfunction and reproductive disorder has been recently discussed in various pieces of research (35).

The rates of spontaneous pregnancy became significantly lower with increasing severity of the disease, whereas the reliance on ART treatment increased with severity. A similar trend has been shown in clinical trials conducted on reproductive medicine patients with the diagnosis of Grade III varicocele (36).

### 4.6 Multivariate analyses indicate oxidative stress as primary determinant

Multiple regression analysis showed that varicocele grade, ROS levels, and MDA content had the highest significant negative impact on spermatogenic outcomes whereas antioxidant activity exhibited beneficial effects. This is in agreement with other multivariate analyses where oxidative stress was determined to be an independent factor influencing male fertility outcome. These correlations further validate oxidative stress as a key treatment target (37, 38).

Zinc, selenium, vitamin C, and vitamin E were found to correlate positively with better semen quality and conception rate. Such results have previously been noted in randomized and observational studies where the administration of antioxidants enhanced the quality of sperms among infertile men (39, 40).

### 4.7 Strengths and limitations of the study and future research directions

The strengths of this study include the use of a large sample size, extensive biochemical analysis, and a multi-faceted statistical approach. The study is however limited by its retrospective nature, limiting its ability to make any causal inferences, as well as self-reporting of dietary information which introduces recall bias. Future prospective and experimental studies are needed to confirm whether oxidative therapies may indeed alter the disease process and result in improved fertility rates.

Despite promising effects, antioxidant therapy is limited by variable bioavailability and pharmacokinetic properties. Many natural compounds exhibit poor systemic absorption and limited distribution to reproductive tissues, including the testes, thereby potentially reducing their clinical efficacy in modulating testicular oxidative stress.

The approximately 24-month follow-up period may be insufficient to fully evaluate long-term fertility outcomes, including natural conception rates and assisted reproductive technology success. Longer-term prospective studies are required to assess these outcomes better.

Certain variables, including perceived stress levels and sleep duration, were measured via self-report and may be subject to measurement bias due to their subjective nature.

## 5 Conclusion

The current observational retrospective study revealed a significant degree-dependent impairment of male reproductive health among infertile men with varicocele. Progressively worsening varicocele grades were invariably found to be positively correlated with poor sperm quality, increased oxidative stress, hormonal disturbances, and decreased fertility rates. Decreased antioxidant and nutritional parameters contributed to lower reproductive success, whereas logistic regression analysis revealed that both varicocele degree and oxidative stress were significant predictors for sperm quality. it can be stated that varicocele negatively influences male fertility via the oxidative, endocrine, and nutritional pathways.

## Data Availability

The raw data supporting the conclusions of this article will be made available by the authors, without undue reservation.
